# Study of whole genome linkage disequilibrium in Nellore cattle

**DOI:** 10.1186/1471-2164-14-305

**Published:** 2013-05-05

**Authors:** Rafael Espigolan, Fernando Baldi, Arione A Boligon, Fabio RP Souza, Daniel GM Gordo, Rafael L Tonussi, Diércles F Cardoso, Henrique N Oliveira, Humberto Tonhati, Mehdi Sargolzaei, Flavio S Schenkel, Roberto Carvalheiro, Jesus A Ferro, Lucia G Albuquerque

**Affiliations:** 1 Faculdade de Ciências Agrárias e Veterinárias, UNESP, Jaboticabal, SP, 14884-000, Brazil; 2 Instituto Nacional de Ciência e Tecnologia - Ciência Animal (INCT- CA), Viçosa, MG, 36570 000, Brazil; 3 Departamento de Nutrição e Produção Animal, Faculdade de Medicina Veterinária e Zootecnia, USP, Pirassununga, SP, Brazil; 4 Centre for Genetic Improvement of Livestock, Department of Animal and Poultry Science, University of Guelph, Guelph, ON, Canada; 5 CNPq Fellowship, National Council of Technological and Scientific Development, 71605-001, SHIS QI 1,Conjunto B, Brasília, DF, Brazil; 6 GenSys Consultores Associados, Porto Alegre, Brazil

**Keywords:** Beef cattle, Genome, Linkage disequilibrium, Genome, Molecular markers

## Abstract

**Background:**

Knowledge of the linkage disequilibrium (LD) between markers is important to establish the number of markers necessary for association studies and genomic selection. The objective of this study was to evaluate the extent of LD in Nellore cattle using a high density SNP panel and 795 genotyped steers.

**Results:**

After data editing, 446,986 SNPs were used for the estimation of LD, comprising 2508.4 Mb of the genome. The mean distance between adjacent markers was 4.90 ± 2.89 kb. The minor allele frequency (MAF) was less than 0.20 in a considerable proportion of SNPs. The overall mean LD between marker pairs measured by r^2^ and |D'| was 0.17 and 0.52, respectively. The LD (r^2^) decreased with increasing physical distance between markers from 0.34 (1 kb) to 0.11 (100 kb). In contrast to this clear decrease of LD measured by r^2^, the changes in |D'| indicated a less pronounced decline of LD. Chromosomes BTA1, BTA27, BTA28 and BTA29 showed lower levels of LD at any distance between markers. Except for these four chromosomes, the level of LD (r^2^) was higher than 0.20 for markers separated by less than 20 kb. At distances < 3 kb, the level of LD was higher than 0.30. The LD (r^2^) between markers was higher when the MAF threshold was high (0.15), especially when the distance between markers was short.

**Conclusions:**

The level of LD estimated for markers separated by less than 30 kb indicates that the High Density Bovine SNP BeadChip will likely be a suitable tool for prediction of genomic breeding values in Nellore cattle.

## Background

Nellore is a beef cattle (Zebu) breed that originated in India. The first specimens of the breed arrived in Brazil at the end of the 18th century and Nellore animals rapidly became the predominant breed in the Brazilian herd [1]. There are about 200 million cattle heads in Brazil and most of them (about 80%) are Zebu animals and their crossbreds [2]. Over the past decades, there has been an increased interest to use genetically evaluated animals in the Zebu population. As a consequence, several genetic evaluation programs of Zebu breeds exist, particularly for Nellore cattle. The main focus of these programs is growth and conformation traits, which are used as selection criteria [3].

The breeding value of animals can be obtained from genomic data by marker-assisted selection covering the whole genome, also called genomic selection [4,5]. Genomic selection explores the linkage disequilibrium (LD) between markers, assuming that the effects of chromosome segments will be the same in the whole population since the markers are in LD with genes that are responsible for expression of the trait (quantitative trait loci, QTL). Therefore, the density of markers should be sufficiently high to guarantee that all QTL are in LD with a marker or with a marker haplotype. The LD maps are important tools for exploring the genetic basis of economically important traits in cattle. Likewise, comparison of LD maps permits to establish the diversity between cattle breeds with different biological attributes and to identify genome regions that were subject to different selection pressures [6].

The two measures most commonly used to evaluate LD between biallelic markers are r^2^ and |D'| [7-10]. These parameters can vary between 0 and 1. A value of |D'| < 1 indicates the occurrence of recombination between two loci, and |D'| = 1 indicates the lack of recombination between two loci. One disadvantage of |D'| is that it tends to be strongly overestimated in small samples and in the presence of rare or low-frequency alleles. The r^2^ parameter represents the correlation between two loci and is preferred in association studies since an inverse relationship exists between r^2^ and the size of the sample needed for the same detection power. Linkage disequilibrium is necessary to detect associations between a QTL and a marker [11].

The LD between markers has been studied in the genome of taurine breeds. In this respect, [12] analyzing 505 SNPs located on chromosome 14 of Holstein cattle, reported moderate levels of LD (r^2^ = 0.2) for markers separated by less than 100 kb. Similar results have been reported by [6] who estimated the LD (r^2^) between 2,670 markers in eight cattle breeds. Villa-Angulo et al., 2009 [13] studied the genomes of 19 taurine and Zebu breeds using a set of 32,826 SNPs. The authors observed that Zebu breeds have a higher proportion of low-frequency alleles and a lower level of LD than taurine breeds. Recently, [14] genotyped 25 Gyr bulls using a panel of 54,000 markers (SNPs) and obtained a mean LD (r^2^) between adjacent markers of 0.21.

The first step necessary to determine the number of markers required for QTL mapping and genomic selection is the quantification of the extent of LD in the cattle genome. Therefore, the objective of the present study was to evaluate LD in Nellore cattle using a high density SNP panel (Illumina High Density Bovine SNP BeadChip®).

## Results and discussion

The results of descriptive statistics of the SNP markers and LD (r^2^ and |D'|) between synthetic adjacent markers obtained for each autosome are shown in Table 1. A total of 446,986 (57.5%) markers met the filtering criteria and were included in the final analysis. This sub-set of markers comprised 2,508.4 Mb of the genome, with a mean distance between markers of 4.90 ± 2.89 kb. The SNPs were uniformly distributed across all autosomes since the marker density was similar for all chromosomes, ranging from 4.9 to 5.2 kb (Table 1). The autosomes differed in size, with BTA25 being the shortest chromosome (42.8 Mb) and BTA1 the longest (158.5 Mb).

**Table 1:** **Summary of the SNP markers analyzed and average linkage disequilibrium** (**r**^**2**^**and** |**D**'|) **between synthetic adjacent markers obtained for each autosome** (**BTA**)^**1**^

**BTA**	**Size (Mb)**	**SNP (n)**	**Mean distance ±SD (kb)**	**Mean r**^**2**^**±SD**	**Median r**^**2**^	**Mean |D'| ±SD**	**Median |D'|**	**Mean MAF ±SD**
1	158.5	28,569	5.0±2.9	0.12±0.22	0.015	0.38±0.34	0.26	0.25±0.13
2	136.8	23,866	5.1±2.9	0.19±0.25	0.077	0.57±0.32	0.60	0.25±0.13
3	121.4	22,592	5.1±2.9	0.19±0.25	0.081	0.57±0.32	0.60	0.25±0.13
4	120.6	20,907	5.1±2.9	0.18±0.24	0.071	0.56±0.32	0.59	0.25±0.13
5	121.1	20,092	5.1±2.9	0.20±0.25	0.086	0.58±0.32	0.62	0.26±0.13
6	119.4	23,500	5.1±2.9	0.19±0.25	0086	0.57±0.32	0.62	0.27±0.13
7	112.6	20,181	5.1±2.9	0.20±0.25	0.082	0.56±0.32	0.60	0.25±0.13
8	113.3	21,667	5.2±2.8	0.20±0.25	0.097	0.59±0.32	0.64	0.26±0.13
9	105.6	20,593	5.1±2.9	0.19±0.25	0.078	0.56±0.32	0.59	0.26±0.13
10	104.2	17,213	5.0±2.9	0.18±0.25	0.064	0.57±0.32	0.55	0.25±0.13
11	107.2	18,477	5.0±2.9	0.19±0.25	0.070	0.56±0.32	0.59	0.24±0.13
12	91.1	15,556	5.1±2.9	0.18±0.24	0.070	0.55±0.32	0.57	0.24±0.13
13	84.2	14,145	5.1±2.9	0.21±0.26	0.094	0.59±0.32	0.65	0.25±0.13
14	83.9	16,909	5.2±2.8	0.18±0.24	0.079	0.56±0.32	0.59	0.26±0.13
15	85.2	14,669	5.0±2.9	0.18±0.24	0.067	0.54±0.32	0.56	0.24±0.13
16	81.7	14,715	5.0±2.9	0.18±0.24	0.072	0.56±0.32	0.59	0.25±0.13
17	75.1	14,108	5.1±2.9	0.19±0.26	0.068	0.55±0.32	0.58	0.24±0.13
18	65.9	11,461	5.0±2.9	0.15±0.22	0.051	0.52±0.31	0.52	0.24±0.13
19	63.9	9,889	5.0±2.9	0.20±0.26	0.088	0.59±0.32	0.63	0.24±0.13
20	71.9	12,734	5.0±2.9	0.18±0.24	0.067	0.54±0.32	0.55	0.25±0.13
21	71.6	12,882	5.1±2.9	0.17±0.24	0.064	0.55±0.32	0.57	0.24±0.13
22	61.3	10,442	5.0±2.9	0.16±0.23	0.061	0.54±0.32	0.55	0.25±0.13
23	52.5	9,369	5.1±2.9	0.17±0.24	0.063	0.55±0.32	0.58	0.25±0.13
24	62.3	11,300	5.1±2.9	0.17±0.23	0.063	0.54±0.32	0.56	0.25±0.13
25	42.8	7,537	5.1±2.9	0.16±0.22	0.058	0.55±0.32	0.56	0.23±0.13
26	51.6	9,503	5.0±2.9	0.17±0.23	0.062	0.54±0.31	0.56	0.24±0.13
27	45.4	7,963	5.0±2.9	0.09±0.18	0.020	0.42±0.31	0.36	0.25±0.13
28	46.2	7,858	4.9±2.9	0.02±0.07	0.003	0.25±0.25	0.16	0.25±0.13
29	51.1	8,289	4.9±2.9	0.003±0.01	0.001	0.12±0.14	0.07	0.24±0.13

SNP: single-nucleotide polymorphism; MAF: minor allele frequency; SD: Standard deviation. ^1^Data in the table is based on the UMD 3.1 assembly.

**Figure 1:**
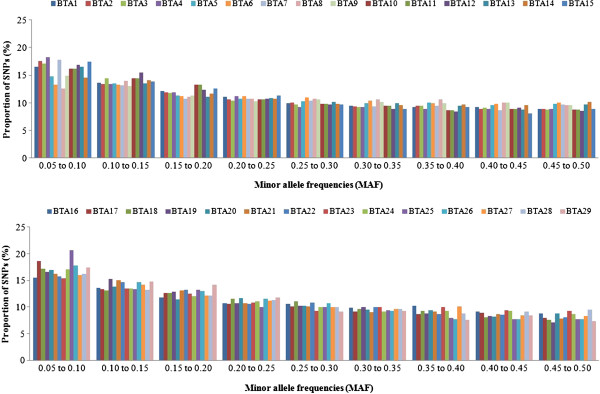
Mean proportion of SNPs for various minor allele frequencies (MAF) calculated for each chromosome (intervals do not include the upper limit).

After filtering of the SNP data, MAF < 0.20 were observed in a considerable proportion of SNPs (Figure 1). Similar results have been reported by [6] and [14] for Zebu breeds. However, the mean MAF obtained in the present study (0.25) was slightly higher than that reported by [15] for Nellore cattle (0.19) and by the Bovine Hapmap Consortium using the Illumina Bovine SNP50K BeadChip for Nellore cattle (0.20) [16]. According to [17], the threshold for MAF affects the distribution and extent of LD. Chromosomes BTA2, BTA4, BTA7, BTA15, BTA17, BTA25 and BTA26 presented a higher proportion of minor alleles (MAF < 0.10), whereas chromosomes BTA6, BTA8, BTA16, BTA22 and BTA23 presented a lower proportion of minor alleles (MAF < 0.10).

All possible SNP pairs on the same chromosome separated by ≤ 100 kb produced 9,254,142 combinations of SNP pairs to estimate LD across the 29 autosomes. The overall mean LD between marker pairs measured by r^2^ and |D'| was 0.17 and 0.52, respectively. Silva et al., 2010 [14] genotyped 25 Gyr sires using a panel of 54,000 markers (SNPs) and obtained a mean LD between adjacent markers measured by r^2^ and |D'| of 0.21 and 0.68, respectively. The present results and those reported in previous studies confirm that the |D'| parameter overestimates LD, especially in cases of low MAF.

The mean LD between adjacent SNPs across autosomes ranged from 0.003 to 0.21 for r^2^ and from 0.12 to 0.59 for |D'| (Table 1). Silva et al., 2010 [14] reported slightly higher values for Gyr cattle, ranging from 0.17 to 0.24 for r^2^ and from 0.60 to 0.72 for |D'|, respectively. Lower levels of LD (r^2^ < 0.16) were estimated for chromosomes BTA1, BTA27, BTA28 and BTA29. This relatively low level of LD obtained for these chromosomes is in contrast to findings previously published for Zebu breeds [6,14]. According to [18], there is a wide variation in autosomal recombination rates, a fact, among others, that leads to marked diversity in the pattern of LD in different genomic regions. However, the results obtained in this study for BTA1, BTA27, BTA28 and BTA29 can probably be attributed to a sampling variation since the number of markers, marker density, mean MAF or proportion of MAF did not differ from the other autosomes studied.

**Figure 2:**
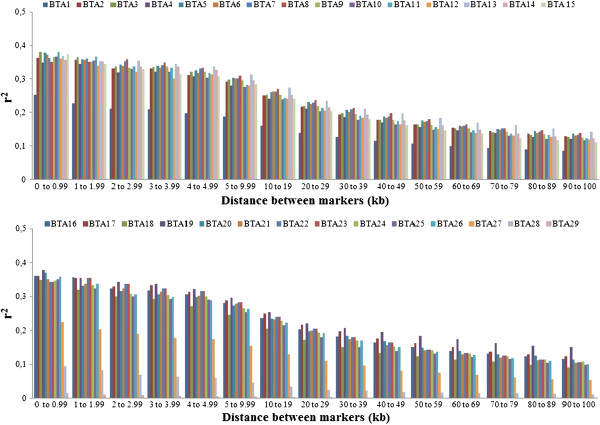
**Mean values of r**^**2 **^**per chromosome according to distance between markers.**

**Figure 3:**
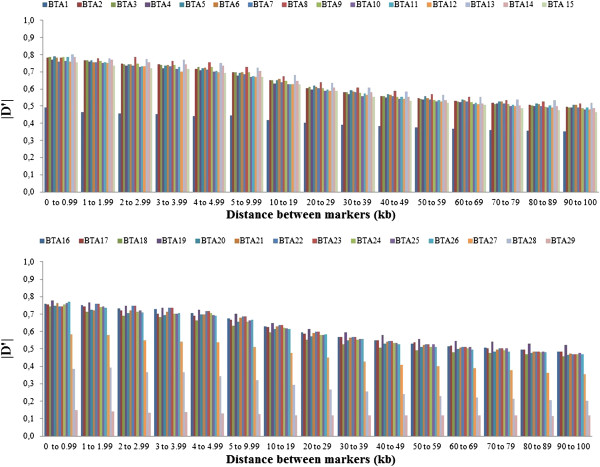
Mean values of |D'| per chromosome according to distance between markers.

To analyze the decline in LD according to physical distance between markers, synthetic SNP pairs were classified into intervals (bins) based on the distance between markers and mean values of r^2^ and |D'| were estimated for each bin per autosome (Figures 2 and 3) and for the whole genome (Table 2). The LD decreased with increasing physical distance between markers (Table 2). In contrast to this clear decrease of LD measured by r^2^, the changes in |D'| indicated a less pronounced decline of LD (Figures 2 and 3). Moderate levels of r^2^ (0.20 to 0.34) were observed at distances < 30 kb. When the distance between markers increased from 30 to 100 kb, the mean r^2^ value decreased from 0.20 to 0.11. A high variability in r^2^ estimates was observed for marker distances of more than 10 kb. Markers showing LD (r^2^) higher than 0.30 and 0.15 had an average spacing of 38.9 and 41.8 kb, respectively. However, not all markers with a spacing of 40 to 50 kb presented an r^2^ value higher than 0.3. For distances of less than 40 kb, the proportion of markers with an r^2^ > 0.15 and > 0.30 ranged from 35 to 57% and from 21 to 42%, respectively. This proportion was lower than that reported by [19] (68.34%) for markers spacing from 0 to 0.1 Mb, who genotyped 821 sires using 5,564 SNPs and the same threshold (0.30) for LD (r^2^). Recently, [20] genotyped 810 Holstein cattle using the Illumina Bovine SNP50K panel and found that, for SNPs separated by less than 100 kb, the proportion of those in LD (r^2^) > 0.25 was 29%.

**Table 2:** **Linkage disequilibrium** (**r**^**2**^**and** |**D**'|) **between pairs** (**N**) **of synthetic SNPs separated by different distances across all autosomes**

**Distance (kb)**	**N**	**Mean r**^**2**^**±SD**	**Median r**^**2**^	**Mean |D'| ±SD**	**Median |D'|**	**% r**^**2**^**> 0.3**^**1**^	**% r**^**2**^**> 0.15**^**1**^	**% |D'| > 0.8**^**1**^
0 - 1	54404	0.34±0.33	0.21	0.72±0.32	0.90	42	57	44
1 - 2	99488	0.32±0.33	0.19	0.70±0.33	0.88	40	55	42
2 - 3	104465	0.31±0.32	0.18	0.69±0.33	0.86	38	54	41
3 - 4	112729	0.30±0.32	0.17	0.68±0.33	0.84	38	53	40
4 - 5	108799	0.29±0.31	0.16	0.67±0.33	0.82	36	51	39
5 - 10	514160	0.27±0.30	0.14	0.64±033	0.77	33	48	37
10 - 20	977946	0.23±0.28	0.10	0.60±0.33	0.68	28	43	34
20 - 30	946535	0.20±0.26	0.08	0.57±0.33	0.61	24	38	30
30 - 40	927788	0.18±0.24	0.07	0.54±0.33	0.57	21	35	28
40 - 50	918536	0.16±0.23	0.06	0.52±0.33	0.53	19	32	26
50 - 60	908630	0.15±0.22	0.05	0.51±0.32	0.50	17	29	24
60 - 70	901928	0.14±0.21	0.05	0.50±0.32	0.48	15	28	23
70- 80	895944	0.13±0.20	0.04	0.48±0.32	0.47	14	26	21
80 - 90	893132	0.12±0.19	0.04	0.47±0.31	0.45	13	24	20
90 - 100	889644	0.11±0.18	0.04	0.47±0.31	0.44	12	23	19

SNP: single-nucleotide polymorphism; SD: Standard deviation. ^1^Percentage of SNP pairs with r^2^ > 0.3, r^2^ > 0.15 and |D'| > 0.8.

Except for autosomes BTA1, BTA27, BTA28 and BTA29, the level of LD (r^2^) was higher than 0.20 for markers separated by less than 20 kb, and higher than 0.30 for markers separated by less than 3 kb. For marker distances higher than 100 kb, the level of LD (r^2^) decreased from 0.11 (100 kb) to 0.05 (1,000 kb) (data not shown). McKay et al., 2007 [6] estimated the LD between all marker pairs (synthetic markers) in eight cattle breeds (*Bos taurus* and *Bos indicus*) and reported a mean LD (r^2^) ranging from 0.15 to 0.20 for a physical distance of 100 kb between adjacent markers.

In the present study, certain autosomes presented higher LD than others. In addition, when autosomes with low levels of LD (r^2^ < 0.17) were excluded (BTA1, BTA27, BTA28 and BTA29), a linear relationship was observed between chromosome length and LD (r^2^), i.e., the level of LD increased with increasing chromosome size. According to [18], recombination rates decrease as the length of the chromosome increases. In a recent study, [10] found no association between chromosome size and level of LD. However, these authors used a *Bos taurus* cattle population and a much lower marker density.

**Figure 4:**
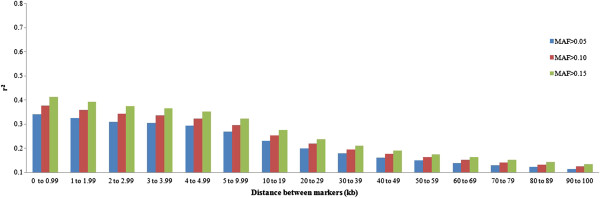
**Mean values of r**^**2 **^**for different thresholds of minor allele frequency (MAF>0.05, MAF>0.10 and MAF>0.15) according to distance between markers.**

**Figure 5:**
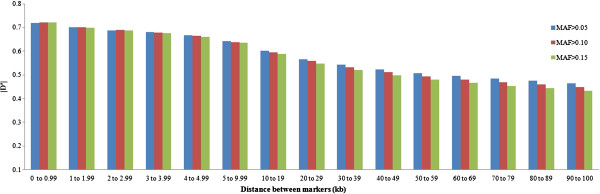
Mean values of |D'| for different thresholds of minor allele frequency (MAF>0.05, MAF>0.10 and MAF>0.15) according to distance between markers.

The use of SNP pairs with low allele frequencies tends to underestimate LD. Polymorphisms with high allele frequencies are thus preferred for a less biased estimation of LD [21]. We therefore analyzed the effect of MAF on the estimates of |D'| and r^2^ (Figures 4 and 5). The LD (r^2^) between markers was higher when the MAF threshold was high (0.15), particularly when the distance between markers was short (Figure 4). Yan et al., 2009 [22], genotyping 632 maize lines using 1,229 SNP markers, showed that the LD (r^2^) between markers increased with increasing MAF threshold, especially in the case of very close SNP pairs (0–10 kb). For adjacent markers (< 10 kb), the |D'| remained unchanged for different MAF thresholds (Figure 5). For more distant markers, the |D'| was lower as the MAF threshold increased. According to [10], the LD measured by |D'| is underestimated as the MAF threshold increases (above 0.25). When LD is determined by |D'|, the denominator in the formula is the product between allele frequencies. Thus, in the case of SNP pairs with low allele frequencies, D' will be divided by a small number, resulting in a large value for |D'| [21]. The results of the present study indicate a considerable variation in the magnitude and pattern of LD in the Nellore genome. As a consequence, two markers that are very close may show a low level of LD, whereas more distant markers may show a higher level of LD than expected. This variation is probably due to different recombination rates between and within chromosomes, heterozygosity, genetic drift, and effects of selection [21].

The level of LD between adjacent markers (distance of less than 30–40 kb) observed in the present study was lower than that reported in other studies on *Bos taurus* cattle and similar to that found in studies using *Bos indicus*. The differences between taurine and indicine breeds decrease for markers separated by 80 to 100 kb. However, it is generally difficult to compare the level of LD obtained in different studies because of differences in sample size, measures of LD, type of markers and marker density, as well as because of the recent history of the population [11]. Nevertheless, differences between indicine and taurine cattle that occurred during the historical process of domestication and selection and as a consequence of the effective size of populations seem to explain the discrepancy in LD at short distances between markers [23]. Another reason is the fact that *Bos indicus* populations present a higher proportion of low-frequency alleles in the HD SNP chip than *Bos taurus* populations which, in turn, influences LD estimates [6,24].

## Conclusions

The level of LD estimated for markers separated by less than 30 kb indicates that the High Density Bovine SNP BeadChip will likely be a suitable tool for prediction of genomic breeding values in Nellore cattle. Further studies investigating the magnitude of LD in a larger sample of animals from this population are needed to confirm the estimates obtained here.

## Methods

Seven hundred and ninety five Nellore bulls born in 2008 and 2009 from 117 sires, which belonged to the three Brazilian beef cattle breeding programs, were used in the present study. This research did not involve humans and the Animal Care and Use Committee approval was not obtained for this study because the data were from an existing database. Genotyping was performed by high density bead array technology using the Illumina Infinium HD Assay® and Illumina HiScan system®. The High Density Bovine SNP BeadChip contains 777,962 SNP markers spread across the genome at a mean distance of 3.43 kb between markers. The HiScan images and genotypes were first analyzed using the Genome Studio® software (Illumina). A total of 1,465 markers were excluded due to unknown genome position and 15,116 markers were monomorphic. For sake of the present study, only autosomal markers with minor allele frequencies (MAF) higher than 0.05, 0.10 or 0.15 were included in the LD analysis. In addition, only markers with a call rate > 0.90 and heterozygote excess < 0.30 were considered. A total of 11,785 markers were excluded because they showed low mean cluster intensity (AB_R, AA_R or BB_R: mean < 0.3).

For DNA extraction, about 5 g of *longissimus dorsi* muscle sample was removed and stored in a 2 ml Eppendorf tube. The tubes were identified with the identification of each animal and then stored in styrofoam boxes in a freezer at −20°C. Next, 25 to 30 mg of muscle tissue specimens were weighed on an aluminum sheet using an analytical balance and transferred to Eppendorf tubes (1.5 to 2 ml). DNA was extracted from the muscle samples using the DNeasy Blood & Tissue Kit (Qiagen GmbH, Hilden, Germany) according to the manufacturer’s instructions.

The LD between two SNPs was evaluated using r^2^ and the absolute value of D'. The r^2^ was calculated as follows:

$$\begin{array}{l} r^{2}=\frac{{\left(\mathrm{freq}.\mathrm{AB}*\mathrm{freq}.\mathrm{ab}-\mathrm{freq}.\mathrm{Ab}*\mathrm{freq}.\mathrm{aB}\right)}^{2}}{\left(\mathrm{freq}.A*\mathrm{freq}.a*\mathrm{freq}.B*\mathrm{freq}.b\right)}\\ \;=\frac{{\left(D\right)}^{2}}{\left(\mathrm{freq}.A*\mathrm{freq}.a*\mathrm{freq}.B*\mathrm{freq}.b\right)} \end{array}$$

where,

D=freq.AB−freq.A*freq.B

and

$$D'=\left\{\begin{array}{l} \frac{D}{\min\left(\mathrm{freq}.A*\mathrm{freq}.b,\mathrm{freq}.a*\mathrm{freq}.B\right)}\;\mathrm{if}\;D>0\\ \frac{D}{\min\left(\mathrm{freq}.A*\mathrm{freq}.B,\mathrm{freq}.a*\mathrm{freq}.b\right)}\;\mathrm{if}\;D<0 \end{array},\;\right\}$$

where *freq*. *A*, *freq*.*a*, *freq*. *B* and *freq*.*b* are the frequencies of alleles A, a, B and b, respectively, and *freq*. *AB*, *freq*.*ab*, *freq*.*aB* and *freq*. *Ab* are the frequencies of haplotypes AB, ab, aB and Ab in the population, respectively. If the two loci are independent, the expected frequency of haplotype AB (*freq*. *AB*) is calculated as the product between *freq*. *A* and *freq*. *B*. A *freq*. *AB* higher or lower than the expected value indicates that these two loci in particular tend to segregate together and are in LD. The measures of LD (r^2^ and |D'|) were calculated for all marker pairs of each chromosome using the SnppldHD software (Sargolzaei, M., University of Guelph, Canada).

Only maternal haplotypes were considered for the estimation of LD measures (r^2^ and |D'|). The exclusive use of maternal haplotypes is a common practice in studies estimating LD when the population consists of half-sib families, as was the case here. The reason is that the pedigree structure leads to the over-representation of paternal haplotypes in the sample since sires have multiple progenies in the dataset, which might increase the frequency of certain haplotypes and consequently overestimate LD [21].

## Abbreviations

LD: Linkage disequilibrium; SNP: Single nucleotide polymorphism; MAF: Minor allele frequency; QTL: Quantitative trait loci.

## Competing interests

The authors declare that they have no competing interests.

## Authors’ contributions

RE and FB participated in the design of the study, performed the genome studio analysis, statistical analysis and drafted the manuscript, AAB participated in the design of the study, helped with the genome studio analysis, statistical analysis and to draft the manuscript, FRPS and DFC participated in the DNA extraction, carried out the molecular analysis and helped to draft the manuscript, DMG, RLT and RE participated in the collection and preparation of the samples, HNO participated in the design of the study and to draft the manuscript, HT helped to draft the manuscript, MS participated in the design of the study, helped with LD analysis and to draft the manuscript, FSS participated in the design of the study, helped with LD analysis and to draft the manuscript, RC participated in the design of the study, helped the genome studio analysis, statistical analysis and to draft the manuscript, JAF carried out the molecular analysis and helped to draft the manuscript, LGA conceived the study and participated in its design and coordination and helped to draft the manuscript. All authors read and approved the final manuscript.
